# Donor Cell Composition and Reactivity Predict Risk of Acute Graft-versus-Host Disease after Allogeneic Hematopoietic Stem Cell Transplantation

**DOI:** 10.1155/2016/5601204

**Published:** 2016-11-14

**Authors:** Darius Sairafi, Arwen Stikvoort, Jens Gertow, Jonas Mattsson, Michael Uhlin

**Affiliations:** ^1^Centre for Allogeneic Stem Cell Transplantation, Karolinska University Hospital, Huddinge, Sweden; ^2^Department of Oncology and Pathology, Karolinska Institutet, Stockholm, Sweden; ^3^Department of Clinical Immunology and Transfusion Medicine, Karolinska University Hospital, Huddinge, Sweden

## Abstract

*Background*. Graft-versus-host disease (GVHD) is a serious complication after allogeneic hematopoietic stem cell transplantation (HSCT). We designed a functional assay for assessment of individual risk for acute GVHD.* Study Design and Methods*. Blood samples were collected from patients and donors before HSCT. Two groups of seven patients each were selected, one in which individuals developed acute GVHD grades II–IV and one in which none showed any clinical signs of GVHD. Peripheral blood mononuclear cells (PBMCs) isolated from donors were incubated in mixed lymphocyte cultures (MLCs) with recipient PBMCs. The cells were characterized by flow cytometry before and after MLC.* Results*. Samples from donors in the GVHD group contained significantly lower frequencies of naïve *γδ* T-cells and T-cells expressing NK-cell markers CD56 and CD94. Donor samples in this group also exhibited lower frequencies of naïve CD95^+^ T-cells compared to controls. After MLC, there were dissimilarities in the CD4/CD8 T-cell ratio and frequency of CD69^+^ T-cells between the two patient groups, with the non-GVHD group showing higher frequencies of CD8^+^ and CD69^+^ T-cells.* Conclusion*. We conclude that a thorough flow cytometric analysis of donor cells for phenotype and allogeneic reactivity may be of value when assessing pretransplant risk for severe acute GVHD.

## 1. Introduction

Although an established treatment, allogeneic hematopoietic stem cell transplantation (HSCT) is associated with several serious side effects. These are generally grouped into three categories: infectious complications, drug toxicity, and graft-versus-host disease (GVHD).

GVHD manifests in an acute and chronic form, each with distinct clinical signs and symptoms. Although both are caused by an immunological reaction between donor-derived lymphocytes and recipient tissue, the pathophysiological mechanisms involved are different [1].

Acute GVHD usually occurs within the first three months after engraftment and it has a more rapid course. The main effector cells are donor T-cells that are transferred with the graft and become activated in response to a proinflammatory milieu and differences in cell-surface antigens. It has been shown that naïve CD4^+^ T-cells may have a particularly important role in the initiation of the GVHD process [2]. Activation of these cells by host-specific antigens elicits an immunological reaction directed against host tissue resulting in local and systemic inflammation. Acute GVHD is also associated with an increased level of immunodeficiency, both directly through the immune-modulatory effect of the ongoing inflammatory process [3] and indirectly due to the immunosuppressive agents used for treatment of this potentially life-threatening condition [4].

With today's methods of tissue typing, it is possible to obtain a high level of matching between donor and recipient regarding major histocompatibility antigens (MHC). Even though this progress has significantly improved the outcome of HSCT, the incidence of acute GVHD has still been as high as 80% in some reports [5]. One reason for this is thought to be the existence of so-called minor histocompatibility antigens. These are polymorphic, non-MHC genes, the end products of which may cause an alloreaction [6].

Severe acute GVHD is often resistant to treatment, and it is a widely accepted consensus that preventive measures significantly improve the chances of a positive outcome, as compared to efforts to treat established disease. For this purpose, a predictive test for GVHD would be of great clinical value, particularly because overtreatment with immunosuppressive agents in itself is associated with serious complications [7, 8]. With today's methods, there is no reliable way of predicting the risk of GVHD with any certainty. The aim of this project was to design a functional* in vitro* test for assessment of pretransplant risk for acute GVHD.

## 2. Material and Methods

### 2.1. Patients and Donors

29 patients and their corresponding sibling donors gave their informed consent and were included in the current study. Inclusion criteria for the study were a HLA matched sibling donor and PBSC as stem cell source, and the apheresis was performed at Karolinska University Hospital. All aspects of this study were reviewed and approved by the regional Ethical Committee in Stockholm (approval number 2008/206-31). Blood samples were collected from patients immediately before the start of conditioning therapy and from the donors in conjunction with harvesting of the peripheral blood stem cell (PBSC) graft. We selected seven recipient/donor pairs where the patients had developed acute GVHD of grades II–IV, within the first three months after HSCT, with manifestation in the skin and the gastrointestinal (GI) tract, with or without liver involvement. Seven control cases were selected from those who had had no signs of GVHD and who had not received any additional immunosuppressive therapy apart from the standard GVHD prophylaxis. The remaining 15 patient/donor pairs were excluded from further studies due to established or suspected acute GVHD grade I. Grading of GVHD was performed according to the Glucksberg criteria [9]. All cases of isolated GI-GVHD were verified by biopsies. All recipients and their sibling donors were tissue-typed by allele-level PCR with sequence-specific primers [10]. Patient-donor pairs were matched regarding HLA-A, HLA-B, HLA-C, HLA-DP, HLA-DQ, and HLA-DR. Details concerning patient characteristics and treatments are given in Table 1. No statistical differences could be observed between the groups for the parameters shown in Table 1.

### 2.2. Antibodies

Fluorescein isothiocyanate (FITC)-, phycoerythrin (PE)-, allophycocyanin (APC)-, BD Horizon™ V450 (V450)-, and PE-Cy5-labelled anti-CD3 (UCHT1); APC-labelled anti-CD27 (L128); FITC-labelled anti-CD19 (HIB19); APC-labelled anti-CD45RO (UCHL1); APC-labelled anti-CD19 (HIB19); FITC-labelled anti-CD56 (MCAM16·2); Alexa Fluor® 700-labelled anti-CD4 (RPA-T4); APC-Cy™7-labelled anti-CD8 (SK1); APC-Cy™7-labelled anti-CD69 (FN50); FITC-labelled anti-CD95 (DX2); PE-Cy7-labelled anti-CD3 (SK7); PE-labelled anti-CD45RA (HI100); FITC-labelled anti-CD28 (CD28.2); FITC-labelled anti-CD94 (HP-3D9); FITC-labelled anti-T-cell receptor (TCR) *αβ* (WT31); PE-labelled anti-TCR *αβ* (T10B9.1A-31); FITC-labelled anti-CD69 (FN50); PE-Cy7-labelled anti-CCR7 (3D12); BD Horizon™ V500 (V500)-labelled anti-CD8 (RPA-T8); and 7-amino-actinomycin D (7-AAD) were purchased from BD Biosciences (Franklin Lakes, NJ). Pacific Blue™-labelled anti-CD107a (LAMP-1) was purchased from Biolegend (San Diego, CA). PE-labelled anti-TCR *γδ* (B1.1) was purchased from eBioscience (San Diego, CA). FITC-labelled anti-TCR pan *γδ* (IMMU510) was purchased from Beckman Coulter (Fullerton, CA). Pacific Orange-labelled anti-CD8 (3B5) was purchased from Invitrogen (Camarillo, CA).

### 2.3. Mixed Lymphocyte Culture

PBMCs were isolated from peripheral blood samples using density-gradient centrifugation (800×g, 20 min; Rotina 420 [Hettich, Beverly, MA, USA] with Lymphoprep [Fresenius Kabi, Oslo, Norway]). They were then cryopreserved at −196°C with 10% DMSO in complete RPMI-1640 medium (Hyclone® [Thermo Fisher Scientific Inc., Waltham, MA, USA] enriched with 10% human AB-serum [Karolinska University Hospital] and 100 mg/mL streptomycin [Gibco, Life Technologies, Paisley, UK]). Donor PBMCs were used as responders in this experiment. The method has been described in detail previously [11]. Briefly, the cells were incubated with 1 *μ*M carboxyfluorescein succinimidyl ester (CFSE; Molecular Probes, Inc. [Thermo Fisher]) in PBS and incubated in 37°C for 10 minutes. The reaction was blocked with PBS containing 10% AB-serum, after which the cells were washed and resuspended in complete RPMI-1640 medium in flat-bottomed 6-well plates at 1 × 10^6^ cells/mL. PBMCs from the corresponding recipients were used as stimulator cells after irradiation with 30 Gy. Responder and stimulator cells were added to the wells in a responder : stimulator ratio of 5 : 1. As controls, we used responder cells that were either untreated, CFSE-stained in complete medium alone, or CFSE-stained in complete medium with 10 *μ*g/mL phytohemagglutinin A (PHA; Sigma-Aldrich, St. Louis, MO, USA). The plates were incubated at 37°C in an atmosphere of 5% CO_2_ for 6 days, after which the cells were harvested, stained, and analysed by flow cytometry. Supernatant from each well was collected and stored at −20°C for later analysis.

### 2.4. Flow Cytometry

Cell-surface staining of thawed, unmanipulated donor PBMCs and responder cells from each MLC condition was performed as described previously [12]. Briefly, cells were incubated with the specified antibodies in PBS for 20 min at 4°C, washed twice in PBS, and incubated with 7-AAD for 15 min at room temperature. Stained cells were analysed on a Beckman Coulter Gallios using Beckman Coulter Gallios acquisition software (Beckman Coulter Inc., Fort Collins, CO) or the BD FACS Canto using BD FACS Diva software (BD Biosciences). The data acquired was analysed with FlowJo software (Tree Star Inc., Ashland, OR). Cells were gated according to fluorescence-minus-one (FMO) samples, which were included in each experiment. Lymphocytes were distinguished from monocytes by their side- and forward-scatter phenotype.

### 2.5. Multiplex Assay

Supernatant samples were analysed for the levels of 26 different cytokines (Eotaxin, G-CSF, GM-CSF, IFN-*α*2, IFN-*γ*, IL-10, IL-12 (p40), IL-12 (p70), IL-13, IL-15, IL-17, IL-1*α*, IL-1*β*, IL-2, IL-3, IL-4, IL-5, IL-6, IL-7, IL-8, IP-10, MCP-1, MIP-1*α*, MIP-1*β*, TNF-*α*, and TNF-*β*). The MILLIPLEX MAP Human Cytokine/Chemokine-Premixed 26 Plex from Millipore [Millipore Corporation, Temecula, CA, USA] was performed according to manufacturer's protocol and as described before [13–15]. The Luminex IS 2.3 software [Luminex Corp., Austin, TX, USA] on the LABScan100 (One Lambda Inc., Canoga Park, CA, USA) was used for analysis.

### 2.6. Statistical Analysis

**Figure 1:**
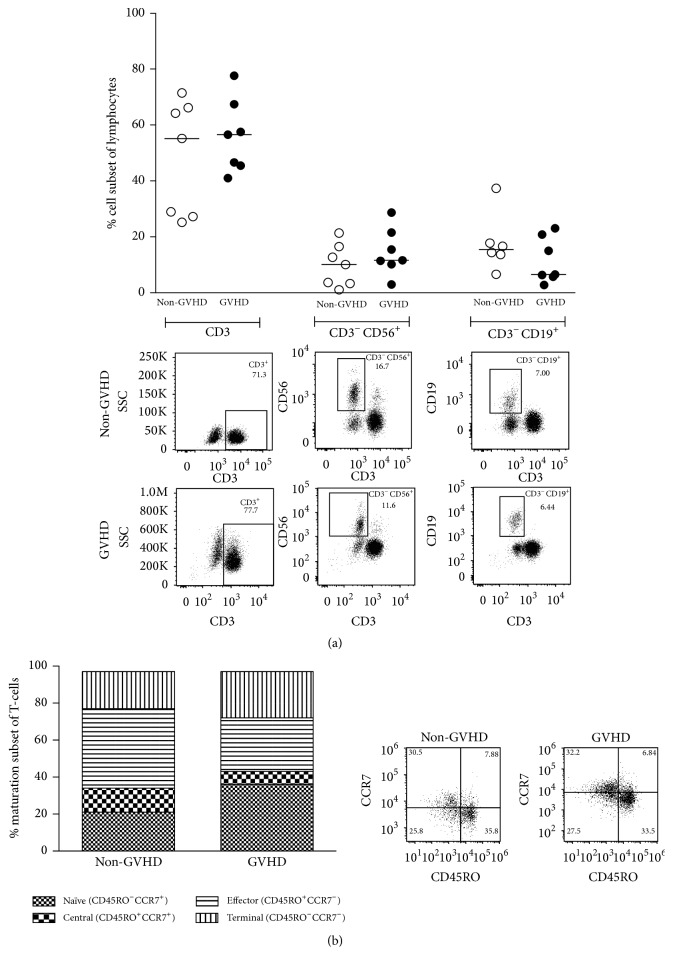
No significant differences between the non-GVHD and GVHD groups regarding major lymphocyte subsets or T-cell maturation subsets in unmanipulated donor samples. Flow cytometry-acquired phenotypic data analysed in blood samples from donors. The data were divided into two groups based on if patients did or did not develop acute GVHD grades II–IV. Each dot represents the cell-subset frequency of one donor and horizontal bars indicate the median of each group. Representative FACS plots are shown below each dot-plot of one non-GVHD and one GVHD patient. (a) Percentages of total T-cells (CD3^+^), NK-cells (CD3^−^CD56^+^), and B-cells (CD3^−^CD19^+^). No differences were observed for these cellular subsets between the non-GVHD and GVHD patient groups. (b) Proportions of T-cell subsets at different maturation states in the total T-cell population, expressed as median percentages. Terminal, terminally differentiated T-cells (CD45RO^−^CCR7^−^); effector, effector memory T-cells (CD45RO^+^CCR7^−^); central, central memory T-cells (CD45RO^+^CCR7^+^); naïve, naïve T-cells (CD45RO^−^CCR7^+^). No differences were observed.

**Figure 2 fig2:**
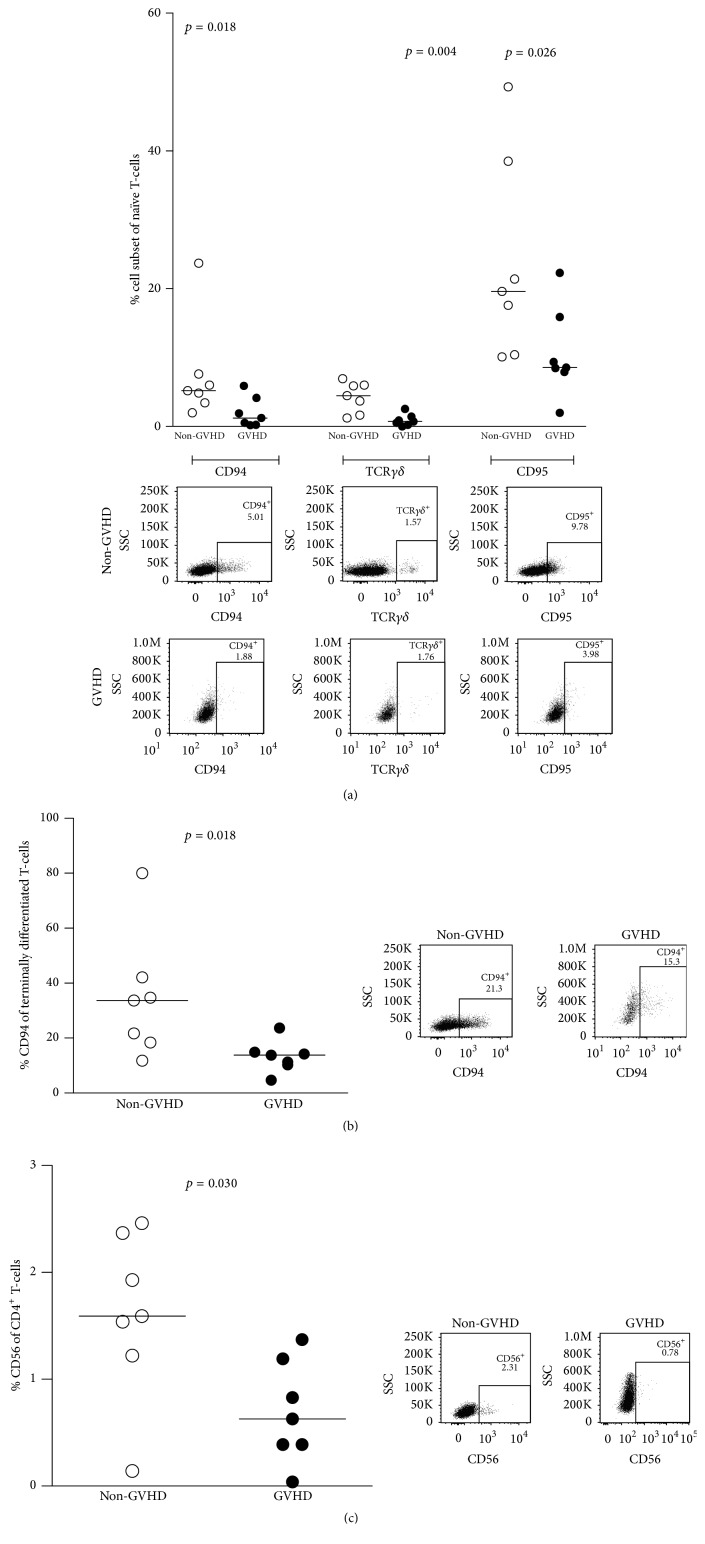
The non-GVHD group had higher frequencies of CD94^+^, TCR*γδ* ^+^, CD56^+^, and CD95^+^ T-cell subsets than the GVHD group in unmanipulated donor samples. Each dot represents the cell-subset frequency of one donor and horizontal bars indicate the median of each group. Representative FACS plots are shown below each dot-plot of one non-GVHD and one GHVD patient. Statistical analysis was done with the Mann–Whitney *U* test. (a) Percentages of CD94^+^, TCR*γδ* ^+^, and CD95^+^ naïve T-cells were increased within the non-GVHD group. (b) CD94 was expressed to a higher degree on terminally differentiated T-cells within the non-GVHD group. (c) Percentages of CD56^+^ CD4^+^ T-cells were increased within the non-GVHD group.

**Figure 3:**
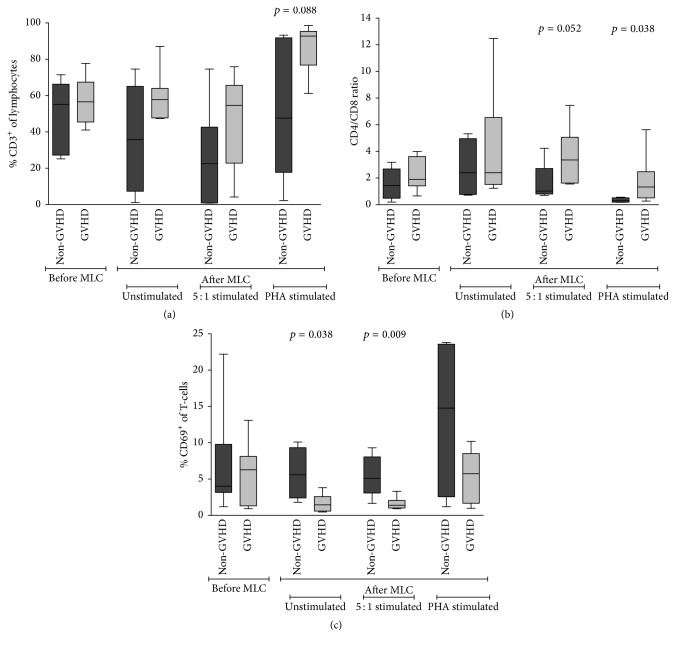
The GVHD group showed dissimilarity in CD4/CD8 T-cell ratios and frequencies of CD69^+^ T-cells when responding donor cells were analysed after MLC. Flow cytometric analysis of responder cells (of donor origin) before and after MLC. Statistical analysis was done with the Mann–Whitney *U* test. (a) Frequencies of T-cells before and after MLC. T-cell frequencies did not differ between the non-GVHD and GVHD groups. (b) CD4/CD8 T-cell ratios before and after MLC. The CD4/CD8 T-cell ratio was similar between the two patient groups before MLC. After MLC, the CD4/CD8 ratio shifted towards an increase of CD4^+^ T-cells and a decrease of CD8^+^ T-cells in the GVHD group. (c) Frequencies of CD69^+^ T-cells before and after MLC. CD69 was expressed more on T-cells of non-GVHD patients after MLC for the unstimulated and 5 : 1 stimulated condition.

Data was analysed and displayed using GraphPad Prism software (GraphPad Software Inc., San Diego, CA) and IBM SPSS Statistics 23 software (IBM, Armonk, NY, USA). Levels and changes in cell-surface markers and levels of soluble factors were compared between the non-GVHD and GVHD groups. The following markers were studied on unmanipulated donor PBMCs: CD3, CD4, CD8, CCR7, CD45RA, CD45RO, CD28, CD69, CD94, CD56, TCR*αβ*, TCR*γδ*, CD95, CD19, and CD27. Responder cells after MLC were analysed for CD3, CD4, CD8, TCR*αβ*, TCR*γδ*, CD45RO, CCR7, CD69, and CD107a. Nonparametric comparisons were performed using the Mann–Whitney *U* test (Table 1; Figures 1 [Fig fig2]–3) and Fisher's exact test (Table 1). Due to sample size limitations, no multivariate analyses were performed. Data are presented as median percentages or as absolute numbers. The number of samples per group is seven unless stated otherwise.

## 3. Results

### 3.1. Properties of Unmanipulated Donor Cells

PBMCs from unmanipulated donor samples were stained for flow cytometric analysis. Acquired phenotypic data were subsequently divided into the two groups “non-GVHD” (*n* = 7) and “GVHD” (*n* = 7) based on patient characteristics after transplantation and analysed for possible differences.

There was no significant difference between the non-GVHD group and the GVHD group regarding frequencies of major lymphocyte populations, that is, total T-cells (median 55.2% versus 56.6%; *p* = 0.535), NK-cells (median 10.1% versus 11.6%; *p* = 0.383), or B-cells (median 15.5% (*n* = 6) versus 6.5%; *p* = 0.295) (Figure 1(a)).

In order to examine the maturation status of T-cells in the grafts, we used the surface markers CD45RO and CCR7. The distribution of the different memory subsets of total T-cells in the two groups is shown in Figure 1(b). No statistically significant differences between the non-GVHD and the GVHD groups were found regarding frequencies of naïve (CD45RO^−^CCR7^+^; median 22.1% versus 43.3%; *p* = 0.165), central memory (CD45RO^+^CCR7^+^; median 12.6% versus 8.7%; *p* = 0.306), effector memory (CD45RO^+^CCR7^−^; median 35.9% versus 28%; *p* = 0.259), or terminally differentiated T-cells (CD45RO^−^CCR7^−^; median 21.7% versus 18.3%; *p* = 0.620). Additionally, no significant differences were seen between the two categories of donors when we analysed total frequencies of CD4^+^ and CD8^+^ T-cells or their individual maturation status.

TCR *γδ*-chains are expressed by a minor population of T-cells [16]. The non-GHVD group had a higher frequency of total TCR *γδ* ^+^ naïve memory T-cells than the GVHD group (median 4.5% versus 0.7%; *p* = 0.004) (Figure 2(a)).

After activation, T-cells transiently express certain NK-cell markers [17]. In this material, CD94^+^ total T-cells were found in higher frequencies in the non-GVHD group for naïve memory T-cells (median 5.2% versus 1.2%; *p* = 0.018; Figure 2(a)) and for terminally differentiated T-cells (median 33.7% versus 13.8%; *p* = 0.018; Figure 2(b)) as compared to the GVHD group. Similarly, CD56 expression on CD4^+^ T-cells was found at higher frequencies in the non-GVHD group (median 1.6% versus 0.6%; *p* = 0.030) (Figure 2(c)).

T-cells expressing the Fas receptor CD95 were observed at significantly higher frequencies in the non-GVHD group in naïve memory T-cells (median 19.6% versus 8.55%; *p* = 0.026; Figure 2(a)) as compared to the GVHD group.

### 3.2. Mixed Lymphocyte Reaction

To evaluate the alloreactive capacity of donor lymphocytes towards recipient cells, a mixed lymphocyte culture (MLC) was performed. By using a multicolor flow cytometry panel, we wanted to detect changes in frequencies of lymphocyte subpopulations of the responding cells and their surface expression of the activation markers CD69 and CD107a after MLC. Ultimately, we wanted to determine whether any changes correlated to the incidence of acute GVHD.

As illustrated in Figure 3(a), no differences in total T-cell percentages could be observed between the patient groups for all three MLC conditions. There was a change in the proportions of CD4^+^ and CD8^+^ T-cells after MLC when we compared the two patient groups. Before the MLC, the CD4/CD8 ratio in both groups was comparable (non-GVHD 1.44 versus GVHD 1.9; *p* = 0.165; Figure 3(b)). At day 6, the CD4/CD8 ratio had changed in the 5 : 1 stimulated samples (non-GVHD 1.02 versus GVHD 3.36; *p* = 0.052) and PHA stimulated samples (non-GVHD 0.30 versus GVHD 1.33; *p* = 0.038). In both settings, the CD4/CD8 ratio skewed towards an increased proportion of CD4^+^ T-cells in the GVHD group as compared to the non-GVHD group (Figure 3(b)).

The frequencies of T-cells positive for early activation marker CD69 were comparable between the two patient groups before MLC (median non-GVHD 4% versus GVHD 6.3%; *p* = 1.0). However, expression of CD69 was higher for total T-cells in the non-GVHD group than in the GHVD group after an unstimulated 6-day incubation (median 5.6% (*n* = 4) versus 1.5% (*n* = 6); *p* = 0.038) and after the 5 : 1 stimulated MLC condition (median 5.1% (*n* = 5) versus 1.4% (*n* = 6); *p* = 0.009; Figure 3(c)). No difference was seen between the two patient groups in CD69^+^ T-cell frequencies after 6-day incubation with PHA.

There was no significant difference in cytokine concentrations in the supernatant of all three conditions after MLC between the two patient groups (data not shown). Additionally, no difference in cell proliferation as measured by CFSE could be observed (data not shown).

## 4. Discussion

Most studies on the effect of graft composition on outcome after HSCT have focused on quantitative differences in doses of total nucleated cells, CD34^+^cells, and T-cells. However, there have been reports indicating that frequencies of minor cell subsets and phenotypic distinctions within these populations could be connected to the alloreactive potential of the graft and may therefore affect clinical outcome [18–25].

In our study we wanted to use multicolor flow cytometry to detect potential differences in frequencies of lymphocyte subsets in donor material before and after* in vitro* MLC in order to predict severe GVHD development. We aimed at creating a robust test that could be used in a potential clinical routine environment. As clinicians and researchers in the field know that grade I acute GVHD can easily be misdiagnosed, we only included patients without any signs of GVHD or with grade II–IV acute GVHD (Table 1). Our results illustrate that flow cytometry on graft material using the most common phenotypic markers is not enough to detect predictive markers for GVHD (Figure 1). Today's use of flow cytometers with >2 lasers in clinical routine enables more in-depth graft analysis.

The biological functions of T-cells that express the *γδ* T-cell receptor (*γδ*TCR) are not fully understood, but existing evidence points towards both proinflammatory and suppressive functions as well as antigen-presenting and cytotoxic capacities [26–28]. In 2001, using an allogeneic rat model, Huang et al. showed that recipients of grafts depleted of *γδ*TCR^+^ T-cells had a higher incidence of acute GVHD than those transplanted with *αβ* T-cell-depleted grafts [23]. It has also been shown that infusion of activated *γδ* T-cells, in the absence of regular T-cells, can promote engraftment without causing GVHD in a MHC-mismatch situation [29]. Other animal studies have shown contradictory results, where *γδ* T-cells appeared to be associated with an increased risk of GVHD [30, 31]. To date, only some clinical studies have addressed the issue of graft *γδ* T-cell content in HSCT [32]. In a report that involved 63 recipients of unrelated peripheral blood stem cell grafts, Pabst et al. [19] found a correlation between high *γδ* T-cell dose and higher incidence of acute GVHD of grades II–IV, while a more recent study has shown contradicting results in a smaller, more heterogeneous cohort of patients [33]. In our material, we found a lower frequency of naïve *γδ* T-cells in the peripheral blood of donors corresponding to patients who later developed acute GVHD of grades II–IV (Figure 2(a)). There could be several possible reasons for the conflicting results. The timing of transfer of the *γδ* T-cells in relation to the allograft has varied between studies. Secondly, if the *γδ* T-cells exert their effect through direct or indirect interaction with other cell types, such as regulatory T-cells and *αβ* T-cells [26, 27, 34, 35], differences in ratios between the infused doses of these cells might lead to different end-results. Lastly, the phenotype of *γδ* T-cell subsets in the graft, particularly their activation and maturation status, could affect their function and ability to survive* in vivo* [36, 37].

Other minor lymphocyte populations that have attracted attention in the context of HSCT are T-cells expressing NK-cell markers [38, 39]. These can be either naturally occurring invariant NKT-cells or* in vitro* activated cytokine-induced killer (CIK) cells. Both cell subsets have been shown to have antitumour and tolerogenic abilities in preclinical studies [40–43]. Invariant NKT-cells have in several studies been shown to be able to reduce the risk for GVHD, whether they were present in high numbers in the graft or given through adoptive transfer [24, 44]. Two independent clinical trials on the use of* ex vivo* expanded allogeneic CIK-cells, as part of treatment for relapsed haematological malignancies after HSCT, have shown response rates comparable to those for regular donor lymphocyte infusions (DLI), but with significantly lower incidence of GVHD [45, 46]. In the present study, we found significantly lower levels of T-cells expressing the NK-cell markers CD56 (on CD4^+^ T-cells) and CD94 (on naïve and terminally differentiated T-cells) in samples from donors before MLC in the GVHD group (Figure 2). If these cells correspond to previously described iNKT-cells, our study confirms that iNKT-cells may indeed have a GVHD-modulatory effect. The exact mechanism for this is not known, but experiments in murine models suggest that these cells have the ability to produce IL-4 and IFN-*γ* upon activation, which would help dampen the immune reaction behind GVHD [25, 47, 48]. Unfortunately, as no NK-cell markers were included in the flow cytometry panel after MLC, it is difficult to speculate on CIK-cells in this study.

Activation-induced cell death (AICD) is important in the regulation of T-cell responses and works as an inhibitory mechanism to prevent uncontrolled activation. AICD may be mediated through the interaction of CD95 (Fas) and CD95 ligand (FasL), which initiates the extrinsic pathway of programmed cell death [49, 50]. The Fas-FasL interaction is of particular importance for establishing peripheral tolerance and it has been shown to have a role in attenuation of GVHD [51–54]. In our material, staining of unmanipulated donor PBMCs for CD95 revealed that samples from the GVHD group contained lower proportions of naïve T-cells that expressed this receptor on their surface (Figure 2(a)). Surface expression of CD95 is greatly increased on T-cells upon activation, while lower expression reflects a more naïve phenotype. This, together with other mechanisms, helps to protect cells from apoptosis in their resting state [55]. It is also in accordance with the widely accepted view that acute GVHD is induced by naïve T-cells, most likely due to their ability to sustain a strong response [56, 57].

Different variations of the MLC have been used to assess the alloreactive capacity of donor cells in the setting of HLA-identical HSCTs, but the correlation to clinical results has varied. In particular, inconsistencies have been observed in matched unrelated transplantations [58–61]. The common principle for these methods is incubation of donor PBMCs with inactivated cells from the recipient followed by quantitative analysis of the helper T-lymphocyte precursor subset. Since these cells appeared to have a high propensity for producing IL-2, an assessment of proliferation and activity was obtained through coincubation with an IL-2-dependent cell line [59]. Multicolor flow cytometry is a more direct and specific approach for quantification and categorization of lymphocyte subpopulations after allogeneic MLC.

By using this sensitive technique, we could detect a shift in CD4/CD8 ratio after MLC between the patient groups. In the 5 : 1 setting, where the cells were exposed to alloantigen presentation, we could observe a trend for an increased proportion of CD4^+^ T-cells in the GVHD group as compared to the non-GVHD group. Moreover, this increased proportion of CD4^+^ T-cells in the GVHD group was also observed in the PHA setting (Figure 3(b)). It would therefore appear that, after stimulation, donor cells from the GVHD group tend to skew more to a CD4^+^ T-cell phenotype than donor cells from the non-GVHD group.

Additionally, we could detect a significant correlation between a decrease in the frequency of CD69^+^ T-cells and incidence of acute GVHD grades II–IV even in this small material (Figure 3(c)). CD69 is traditionally seen as one of the earliest markers emerging after activation of T-cells, though it appears to also be highly expressed by lymphocytes at mucosal sites of the human body [62, 63]. The counter-intuitive observation in the present study, that is, low frequency of activated T-cells correlating with GVHD, might be due to an increased downregulation of CD69 in alloreactive T-cells after 6 days of activation. CD69 is known to be transiently expressed, with an expression as early as 2 hours after stimulation, but with a quick decline of expression after more than 24 hours [64]. Thus, low levels of CD69^+^ T-cells after MLC might serve as an indicator of increased alloreactivity and risk for incidence of acute GVHD.

It was surprising to find no differences in cytokine production between the patient groups. It is possible that the 6-day MLC leads to a saturation of the system due to overstimulation. Periodic sampling during the MLC would have been elucidating. Additionally, the flow cytometry panel could be expanded to include more cellular markers to identify frequencies of other cellular populations that might play important roles in this context, such as regulatory T-cells, myeloid-derived suppressor cells (MDSCs), and mucosal-associated-invariant T-cells (MAITs). Recent studies suggest that these may play critical roles in GVHD development after HSCT and therefore warrant more extensive studies [65–69].

In conclusion, our results indicate that phenotypic analysis of the donor lymphocyte subpopulations before transplantation can yield predictive information of clinical relevance. We found a positive correlation between lower frequencies of donor T-cells expressing TCR *γδ* and NK-cell markers on the one hand and incidence of acute GVHD of grades II–IV on the other hand. We have also shown that a distinct relative decrease in CD69^+^ T-cells in a flow cytometry-based allogeneic MLC can be predictive of acute GVHD.

We recognise the fact that the sample groups in this pilot study are small and not perfectly matched for some clinical parameters, for example, diagnosis or conditioning regimen. Our results, therefore, need to be verified in a larger prospective trial. Nevertheless, the findings presented here may be of value in pretransplant risk assessment and they may enable the use of a more individualized prophylactic strategy.

## Figures and Tables

**Table 1:** Patient and donor characteristics.

Criteria	Group 1 (non-GVHD)	Group 2 (GVHD)
*Patients*, *n*	7	7
*Recipient *		
Age at HSCT (median (range))	47 (30–58)	51 (36–64)
Gender, *n* (female/male)	3/4	3/4
*Diagnosis, n*		
AML	1	3
ALL	2	0
CLL	1	0
MDS/MPD	3	2
Solid tumour	0	2
*Donor*		
HLA-identical sibling, *n*	7	7
Age at HSCT (median (range))	40 (31–67)	54 (43–68)
*Conditioning regimen*, *n*		
Cy + fTBI	2	0
Cy + Bu	2	4
Flu + Bu	2	1
Flu + Cy	0	2
Cy + fTBI + Flu	1	0
*Antithymocyte treatment*, *n*		
ATG	1	0
Campath	1	0
*GVHD prophylaxis*, *n*		
CsA + MTX	7	5
FK + RAPA	0	2
*Graft source*, *n*		
PBSCs	7	7
*Cell dose, median (range)*		
Total nucleated cells (×10^8^/kg)	8.6 (7.5–21.6)	14.8 (5.0–24.5)
CD34^+^ cells (×10^6^/kg)	8 (4.7–11)	9.1 (4.3–9.6)

AML, acute myeloid leukaemia; ALL, acute lymphoblastic leukaemia; ATG, anti-T-cell globulin; Bu, busulfan; CLL, chronic lymphocytic leukaemia; CsA, cyclosporine A; Cy, cyclophosphamide; FK, tacrolimus; Flu, fludarabine; fTBI, fractionated total body irradiation; GVHD, graft-versus-host disease; HLA, human leukocyte antigen; MDS, myelodysplastic syndrome; MPD, myeloproliferative disorders; MTX, methotrexate; *n*, number; PBSC, peripheral blood stem cell; RAPA, sirolimus. Statistical analysis was done with the Mann–Whitney *U* test and Fisher's exact test.
